# Rumen Microbiome and Metabolome of Tibetan Sheep (*Ovis aries*) Reflect Animal Age and Nutritional Requirement

**DOI:** 10.3389/fvets.2020.00609

**Published:** 2020-09-02

**Authors:** Huan Li, Qiaoling Yu, Tongtong Li, Liye Shao, Ming Su, Huakun Zhou, Jiapeng Qu

**Affiliations:** ^1^School of Public Health, Lanzhou University, Lanzhou, China; ^2^Key Laboratory of Restoration Ecology for Cold Regions in Qinghai, Xining, China; ^3^Department of Applied Biology, College of Biotechnology and Bioengineering, Zhejiang University of Technology, Hangzhou, China; ^4^Key Laboratory of Health Aquaculture and Product Processing in Dongting Lake Area of Hunan Province, Zoology Key Laboratory of Hunan Higher Education, Hunan University of Arts and Science, Changde, China; ^5^Central South Inventory and Planning Institute of National Forestry and Grassland Administration, Changsha, China; ^6^Key Laboratory of Adaptation and Evolution of Plateau Biota, Northwest Institute of Plateau Biology, Chinese Academy of Sciences, Xining, China

**Keywords:** rumen microbiome, metabolomics, SCFA, essential amino acids, age

## Abstract

The rumen microbiota plays an important role in animal functional attributes. These microbes are indispensable for the normal physiological development of the rumen, and may also convert the plant polysaccharides from grass into available milk and meat, making it highly valuable to humans. Exploring the microbial composition and metabolites of rumen across developmental stages is important for understanding ruminant nutrition and metabolism. However, relatively few reports have investigated the microbiome and metabolites across developmental stages in ruminants. Using 16S rRNA gene sequnecing, metabolomics and high-performance liquid chromatography techniques, we compared the rumen microbiota, metabolites and short chain fatty acids (SCFAs) between lambs and sub-adult Tibetan sheep (*Ovis aries*) from Qinghai-Tibetan Plateau. Bacteroidetes and Spirochaetae were enriched in sub-adult sheep, while Firmicutes and Tenericutes were more abundant in young individuals. The sub-adult individuals had higher alpha diversity values than those in young sheep. Metabolomics analysis showed that the content of essential amino acids and related gene functional pathways in rumen were different between the lambs and sub-adult population. L-Leucine that participates in valine, leucine and isoleucine biosynthesis was more abundant in the lambs, while phenylethylamine that takes part in phenylalanine metabolism was more enriched in the sub-adults. Both rumen microbial community structures and metabolite profiles were impacted by age, but rumen SCFA concentration was relatively stable between different age stages. Some specific microbes (e.g., *Clostridium* and Ruminococcaceae) were positively associated with L-Leucine but negatively correlated with phenylethylamine, implying that rumen microbes may play different roles for metabolite production at different ages. Mantel test analysis showed that rumen microbiota was significantly correlated with metabolomics and SCFA profiles. Our results indicates the close relationship between microbial composition and metabolites, and also reveal different nutritional requirement for different ages in ruminants, thus having important significance for regulating animal nutrition and metabolism by microbiome intervention.

## Introduction

Ruminants have a close relationship with their symbiotic microorganisms in the digestive tract. These microbes include bacteria, archaea, fungi, viruses, and protozoa (1), which provides many ecosystem functional services on hosts. A typical example is the rumen of ruminants, and the microbial communities in this organ are able to convert those indigestible structural plant polysaccharides from grass into available compounds for animals to help hosts acquire enough energy and promote animal growth (2). This ecological process is very important to human beings because it can convert solar energy stored in plant materials into available food, such as meat and milk. Thus, understanding the composition and function of rumen microbiome is pivotal for improving the productivity of ruminant digestive system.

During the infancy of ruminants, the rumen is not yet developed and lack fermentation function for grass when animals are still suckling milk. One important reason is that the rumen wall papillae, as a region for nutritional absorption, is not mature (3). Some reports have revealed the rapid colonization of aerobic and facultative anaerobic microorganisms at birth (4). With the maturation of animals, foreign anaerobic microorganisms from animal's parents and their surrounding environment gradually replace those aerobic and facultative anaerobic taxa and keep a constant level at between 6 and 8 weeks of age (5). For example, the anaerobic cellulolytic bacteria (*Ruminococcus* and *Fibrobacter*) in the bovine had a low abundance in the newborn (1–3 days) individuals, while had higher abundance in the adult sheep and maintained a relatively constant level (5). When animal reaches sub-adult or adulthood stages, these rumen microbes ferment food, especially those indigestible plant cellulose to produce short chain fatty acids (SCFAs), microbial cell protein, ammonia and other unknown metabolites (1). Although rumen bacterial communities have been investigated from birth to adulthood, there is still lack of information on microbial functional taxa and metabolites. More importantly, the relationship between rumen microbiota and metabolome remains largely unknown.

Metabolomics is one of latest popular “omics” science field based on high-throughput methods, such as liquid chromatography and mass spectrometry (LC/MS), which can extract many metabolites from samples and uncover the metabolic phenotypes in humans (6), animals (7), plants (8), and microbes (9). Metabolomics has opened new insights in animal nutrition research, and has revealed some metabolic pathways and metabolic biomarkers in ruminants in response to food and environmental stress. For example, the goats with high-grain feeding had increased levels of several toxic and inflammatory compounds, such as tryptamine, endotoxin, tyramine, and histamine (7). In addition, LC-MS analysis unraveled that 13 potential metabolic biomarkers associated with carbohydrate, amino acid and lipid metabolism were enriched in dairy cows under controlled heat stress, indicating the metabolic effects of environmental stress on ruminants (10). However, relative few reports have described metabolomics features and related metabolic pathways under different age stages in ruminants.

Tibetan sheep (*Ovis aries*) are one of the most widely distributed and numerous livestock on the Qinghai-Tibetan Plateau, as their population size reaches more than 50 million (11). They provide abundant milk, meat and income for local Tibetan herdsman by converting the plant polysaccharides into available products. Several reports have uncovered the microbiota composition and gene functions in the digestive tract of ruminants on the Qinghai-Tibetan Plateau (12), but there is still paucity of information on the rumen microbiome and metabolites in the Tibetan sheep. In this study, we performed microbiome, metabolome and short chain fatty acid (SCFA) analysis for rumen contents in lambs and sub-adult Tibetan sheep. We mainly addressed the following questions. (1) Whether the microbiomes of Tibetan sheep were different between the young and sub-adult Tibetan sheep? (2) Which rumen metabolites (including SCFAs) and functional pathways were associated with age? (3) Was there a correlation between microbiome and metabolome or SCFAs?

## Materials and Methods

### Animal Feeding and Sampling

Rumen contents were collected between February and July from the Haibei Demonstration Zone of Plateau Modern Ecological Animal Husbandry (100.9518°E, 36.9181°N). After the sheep were anesthetized using diethylether and dissected, we obtained a total of 12 rumen samples from 1-month-old (the lamb group, abbreviated as ES; *N* = 6) and 6-month-old Tibetan sheep (sub-adult group, abbreviated as SS; *N* = 6) in the same cohort. Three portions (~5 g per portion) of the contents from the anterior, middle and posterior parts of the rumen were taken and mixed well-before sample collection. These Tibetan sheep were all males. The sub-adult individuals were grazed on pasture (The main grass included *Kobresia humilis, Oxytropis ochrocephala, Poa* sp.) on the Qinghai-Tibet Plateau, and supplied commercial feed#8876 (Yongxing Ecological Agriculture and Animal Husbandry Development Co., Ltd. in Mengyuan County) at dusk. The main nutritional components of this commercial feed include crude protein ≥ 16%, crude fat ≥ 3%, crude fiber ≤ 8.0%, and crude ash ≤ 9.0%. The daily feed intake of sub-adult individuals was 4.75 ± 0.32 kg. The lamb individuals were mainly fed milk, and also ate a small amount of the grass (~0.72 ± 0.0.07 kg) and commercial feed (~0.54 ± 0.0.12 kg) mentioned above. Drinking water was freely available to these Tibetan sheep. The body weight of lamb and sub-adult groups was 15.75 ± 5.90 and 26.35 ± 4.01 kg, respectively. After collection, the ruminal contents were immediately divided into three parts on ice for the following microbiome, metabolome and short chain fatty acid (SCFA) analysis, and were temporarily kept in −20°C portable refrigerator in field. Finally, all samples were transferred to our lab within 24 h and stored at −40°C refrigerator.

The experimental protocols in current study were allowed by the Animal Welfare and Ethics Committee of Lanzhou University. The related experimental methods and procedures strictly followed the guidelines of the aforementioned institutions.

### Microbiome Sequencing and Bioinformatic Analysis

The Soil Ezup DNA extraction kit (Sangon Biotech, China) was applied to extract total microbial DNA from the ruminal contents according to the product manual. Thereafter, Qubit 2.0 Fluorometer (Invitrogen, Carlsbad, CA) was used to detect the concentration of DNA samples. The V3-V4 regions of 16S rRNA gene were amplified using the primer pairs 341F(CCTACGGRRBGCASCAGKVRVGAAT) and 806R (GGACTACNVGGGTWTCTAATCC) (13). PCR amplification was performed in duplicate. The detailed procedures of PCR amplification and MiSeq (Illumina, San Diego, CA, USA) sequencing have been recorded previously (13).

Microbiome data analysis was mainly performed using QIIME Pipeline-Version 1.9.0 (http://qiime.org/scripts/index.html) from our Microbiome and Bioinformatics Platform in School of Public Health, Lanzhou University. The detailed analysis methods and procedures were described in our previous reports with slight modification (14–17). In brief, the original paired-end reads produced by MiSeq sequencer were assembled using the FLASH-1.2.8 software (18). Those sequences containing N, with base length < 200 bp, or average sequencing quality score < 30 were filtered. After removing chimeras, the remaining sequences were clustered into operational taxonomic units (OTUs) using VSEARCH-1.9.6 at 97% sequence similarity (19). Those OTUs with only one sequence across all samples were excluded. The longest reads for each OTU was picked as the representative sequence. These representative reads were further aligned against the Silva 123 database using PyNAST (20). Taxonomic arrangement of these representative OTUs was based on the Ribosomal Database Project (RDP) classifier with 80% threshold (21) at different categorical level. Those taxa that were not classified as “bacteria” were removed in OTU table.

To minimize the impact of sequencing depth for different samples, each sample was rarefied to 25,131 sequences. Alpha diversity values, including phylogenetic diversity and observed OTUs, were calculated. Beta diversity indices, including unweighted and weighted UniFrac distance matrices (both consider phylogentic relationship between microbes), were calculated using QIIME Pipeline. Unweighted UniFrac distance matrice is based on the absence or presence of OTUs, while weighted UniFrac is dependent on the relative abundance of OTUs (22). Principal coordinates analysis (PCoA) plots based on the above distance matrices were visualization using Origin 2018 (Originlab, Northampton, USA).

### Metabolomic Measurement

One hundred mg ruminal contents were transferred into 5 mL centrifuge tubes, and then 500 μ L ddH_2_O (4°C) were added into the tubes. The mixture was thoroughly vortex-mixed for 60 s. Thereafter, 1,000 ul of methanol (pre-cooled at −20°C) was added into the samples, and the mixed liquids were shaken for 30 s. Thereafter, we further placed the tubes into an ultrasound machine at room temperature for 10 min, and then stew for 30 min on the ice. The samples were centrifuged for 10 min at 14, 000 rpm 4°C, and then 1.2 mL supernatant was transferred into a new centrifuge tube. Samples were further blow-dried by vacuum concentration. Thereafter, samples were dissolved using 400 μl methanol aqueous solution (1:1, 4°C), and underwent 0.22 μm membrane for filtration. For the quality control (QC) samples, 20 μL of prepared samples were extracted and mixed. These QC samples were used to monitor deviations of the analytical results from these pool mixtures. Finally, samples were ready for LC - MS (Waters, Milford, MA, USA) detection. More detailed methods for LC-MS procedures have been described in the previous report (23).

The original data obtained was converted into mzXML format using Proteowizard software (v3.0.8789). Then the metabolomic data underwent peaks identification, filtration and alignment using the XCMS package in R (v3.3.2). In order to compare data of different magnitude, peak area was normalized for further statistical analysis (24).

### Short Chain Fatty Acid Measurement

Short chain fatty acid (SCFA) profiles of ruminal content samples were measured using an Agilent 1100 series high-performance liquid chromatography (HPLC) system (Agilent Technologies, Santa Clara, CA, USA). We measured the concentration of acetate, propionate, butyrate, isobutyrate, valerate, isovalerate, and hexanoate using an Alltech IOA-2000 organic acid column. The detailed procedures for measuring SCFAs have been described previously (25).

### Statistical Analysis

To test the differences of the alpha diversity indices (phylogenetic diversity and observed OTUs) of ruminal microbiomes, Mann–Whitney *U*-tests were used. Permutational multivariate analysis of variance (PERMANOVA) analysis based on the unweighted and weighted UniFrac distance matrices was used to evaluate whether the community structures were significantly distinct between groups using the procedure “Adonis” in the R “vegan” package. In order to identify significant taxonomic differences of rumen microbiota between groups, we used LEfSe analysis at phylum, genus and OTU level. This method considers both biological and statistical significance of data. Linear discriminant analysis (LDA) effect size (LDA > 2) was applied to assess the magnitude scale of the effect of differentially abundant taxa. This analysis was performed at Galaxy module of LEfSe (http://huttenhower.sph.harvard.edu/galaxy/).

For metabolomic data, the detailed analysis methods were described in Li et al. (23). Briefly, the Soft Independent Modeling of the Class Analogy (SIMCA)-P (Version 11.0) was used to perform the principal component analysis (PCA) and partial least squares-discriminant analysis (PLS-DA). Those variables with VIP (Variable Importance in the Projection) > 1 that play major roles were chosen for the following analysis. One-way ANOVA was used to identify the significantly different metabolites (*P* < 0.05) between the groups ES and SS. The identification of these compounds was based on their MS/MS spectra, online databases and literature. Subsequently, the related metabolic pathways of the above different metabolites were analyzed based on Hypergeometric test using the online software MetaboAnalyst (www.metaboanalyst.ca). The impacting factor plots of KEEG (Kyoto Encyclopedia of Genes and Genomes) based on those different metabolites were visualized.

One-way analysis of variance (one-way ANOVA) with Tukey's *post-hoc* test in SPSS13.0 was used to analyze the differences of SCFA concentration of sheep ruminal contents between young and sub-adult groups. The spearman correlation analysis between rumen bacterial genera and SCFAs, differential metabolites was also performed using SPSS13.0. Heatmap2 was used to visualize the correlation results. In addition, mantel test was used to detect the relationship between rumen microbiota and metabolic profile or SCFAs using the “mantel” procedure in the R vegan package.

### Nucleotide Sequence Accession Numbers

All the original 16S rRNA gene data in this study were submitted to the European Nucleotide Archive, and were available by accession NO. PRJEB36249 (http://www.ebi.ac.uk/ena/data/view/PRJEB36249).

## Results

### Overall Rumen Microbiota Composition

**Figure 1:**
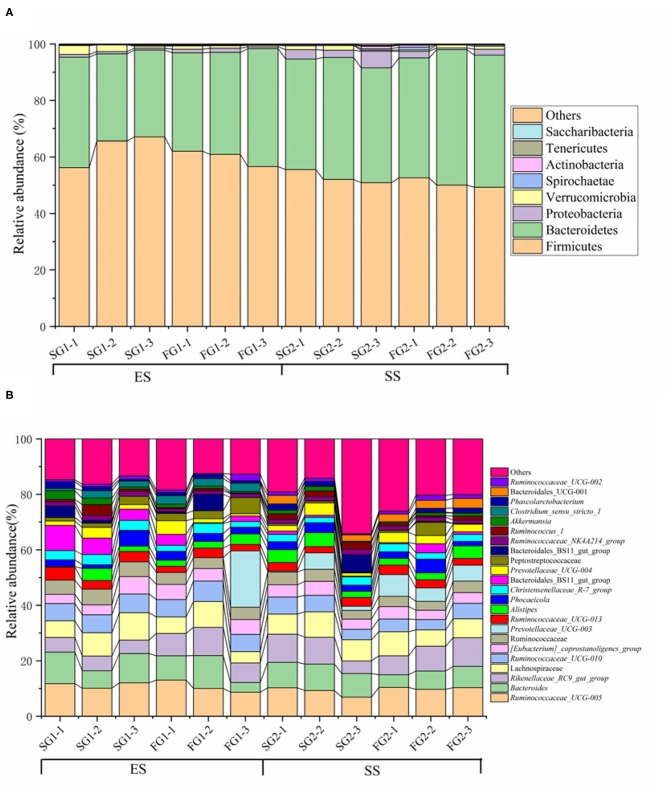
The rumen microbiome composition at phylum **(A)** and genus **(B)** level. Only those with mean relative abundance more than 0.1% (for phyla) or 1% (for genera) are shown.

We obtained 698,333 original paired-end reads across all samples (Mean = 58,194, SD = 5,550) from MiSeq sequencer. A total of 800 unique OTUs were detected in the Tibetan sheep rumen across all samples using VSEARCH clustering. By taxonomic classification, we found that the rumen microbiota was dominated by Firmicutes (mean relative abundance = 56.57%), Bacteroidetes (39.46%), Proteobacteria (1.88%) and Verrucomicrobia (1.24%), followed by other rare phyla (mean relative abundance <1%), such as Spirochaetae, Actinobacteria, Tenericutes and Saccharibacteria. At genus level, five most abundant genera were *Ruminococcaceae_UCG-005* (10.22%), *Bacteroides* (8.18%), *Rikenellaceae_RC9_gut_group* (7.58%), two unknown genus from Lachnospiraceae (7.42%) and *Ruminococcaceae_UCG-010* (5.77%). The rumen microbial composition of each sample was visualized in Figure 1.

### The Difference of Rumen Microbial Composition and Diversity Between Young and Sub-adult Tibetan Sheep

**Figure 2:**
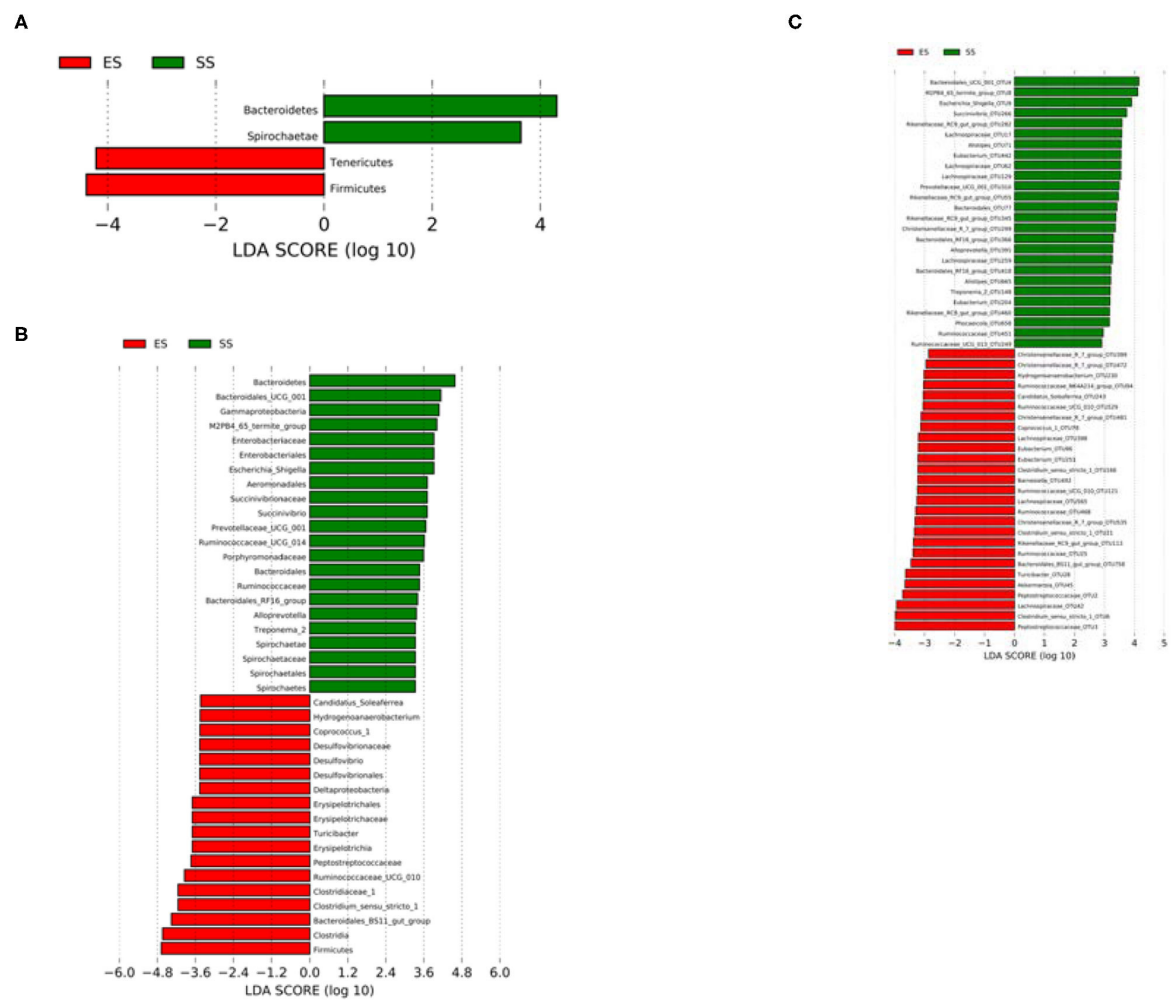
Differences in the rumne microbiota between juvenile and sub-adult Tibetan sheep. Linear discriminant analysis (LDA) effect size (LEfSe) results show that bacterial phyla **(A)**/genera **(B)**/OTUs **(C)** were significantly different in abundance between the two age stages.

**Figure 3:**
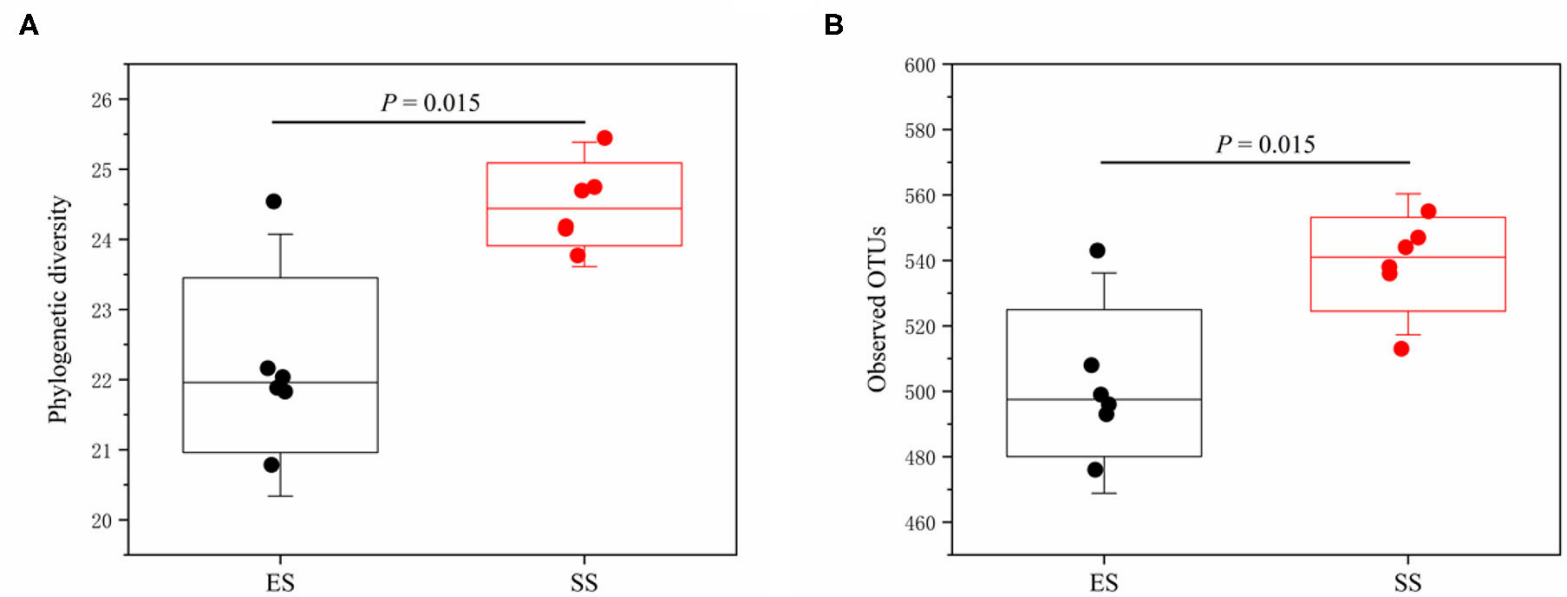
The comparisons of phylogenetic diversity **(A)** and observed OTUs **(B)** of the rumen microbiota between juvenile and sub-adult Tibetan sheep. Mann–Whitney *U*-test was used to test the differences between groups. Significant difference is indicated by *P* < 0.05.

**Figure 4:**
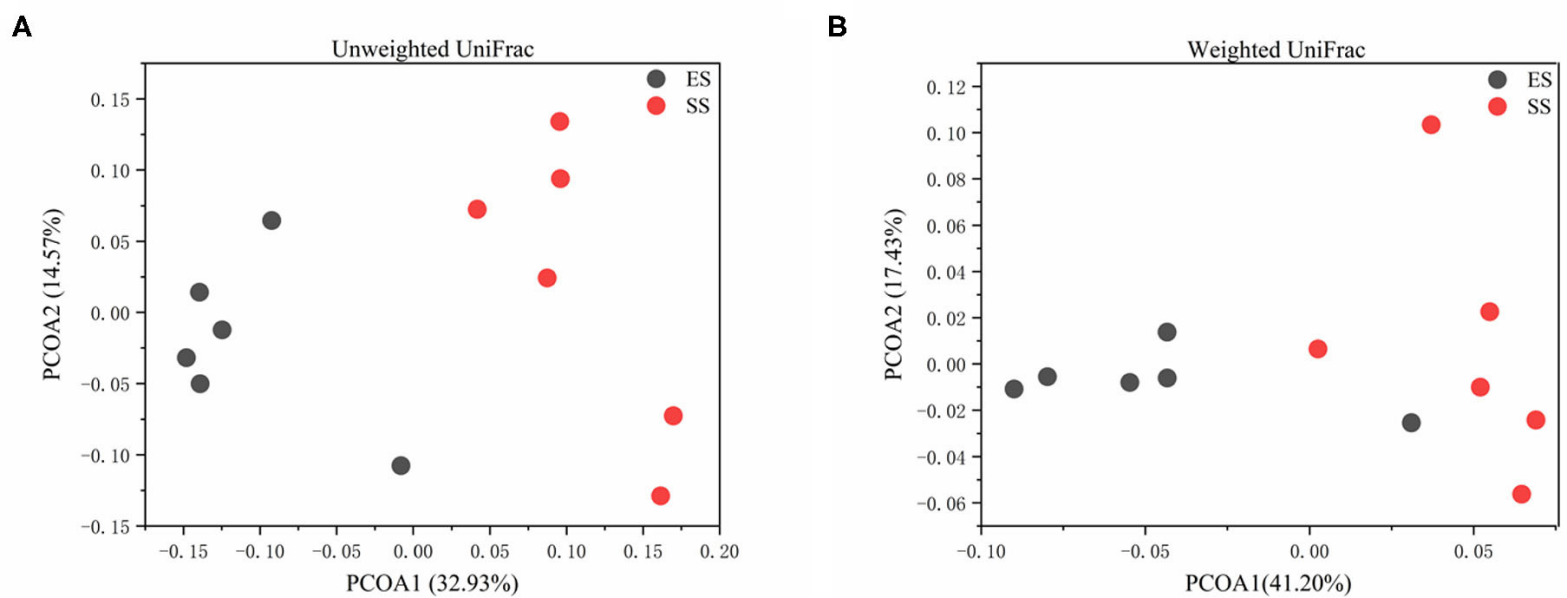
The principal coordinate analysis of rumen microbiota based on unweighted **(A)** or weighted UniFrac **(B)** dissimilarities between juvenile and sub-adult Tibetan sheep.

Using LefSe analysis, we compared the significant different bacteria taxa between developmental stages of Tibetan sheep at phylum, genus and OTU level (Figure 2). We found that Bacteroidetes and Spirochaetae were more enriched in the sub-adult individuals, while Firmicutes and Tenericutes were more abundant in the young sheep. At genus level, a total of 40 genera were significantly different between the young and sub-adult sheep. Among these genera, the ES (or lamb) group showed a higher abundance for 18 genera, such as *Clostridium_sensu_stricto_1, Turicibacter* and *Ruminococcaceae_UCG_010*. The SS (or sub-adult) group was enriched by 22 genera, including *Escherichia_Shigella, Prevotellaceae_UCG_001, Succinivibrio* and *Treponema_2*. At OTU level, a total of 27 OTUs were more abundant in the ES group, these OTUs were mainly affiliated with *Clostridium_sensu_stricto_1*, Ruminococcaceae, *Barnesiella, Akkermansia* and *Eubacterium*. In contrast, 26 OTUs showed a higher abundance in the SS group, these OTUs mainly belonged to Lachnospiraceae, *Succinivibrio*, Rikenellaceae_RC9_gut_group, Ruminococcaceae, *Eubacterium* and Bacteroidales. In addition, we compared the alpha diversity values between the ES and SS groups. The rumen microbiota of sub-adult sheep had higher phylogenetic diversity and observed OTUs (both *P* < 0.05, Figure 3) compared with those in young individuals. For beta diversity, PCoA analysis showed that the young sheep had clear structural separation of microbial communities based on the unweighted or weighted UniFrac dissimilarities (Figure 4). PERMANOVA analysis confirmed that these two groups had significantly different community structures (*R* ^2^ = 0.294, *P* = 0.002 for unweighed UniFrac dissimilarity; *R*^2^ = 0.312, *P* = 0.008 for weighed UniFrac dissimilarity).

### The Difference of Rumen Metabolomics Profiles Between Young and Sub-adult Sheep

**Figure 5:**
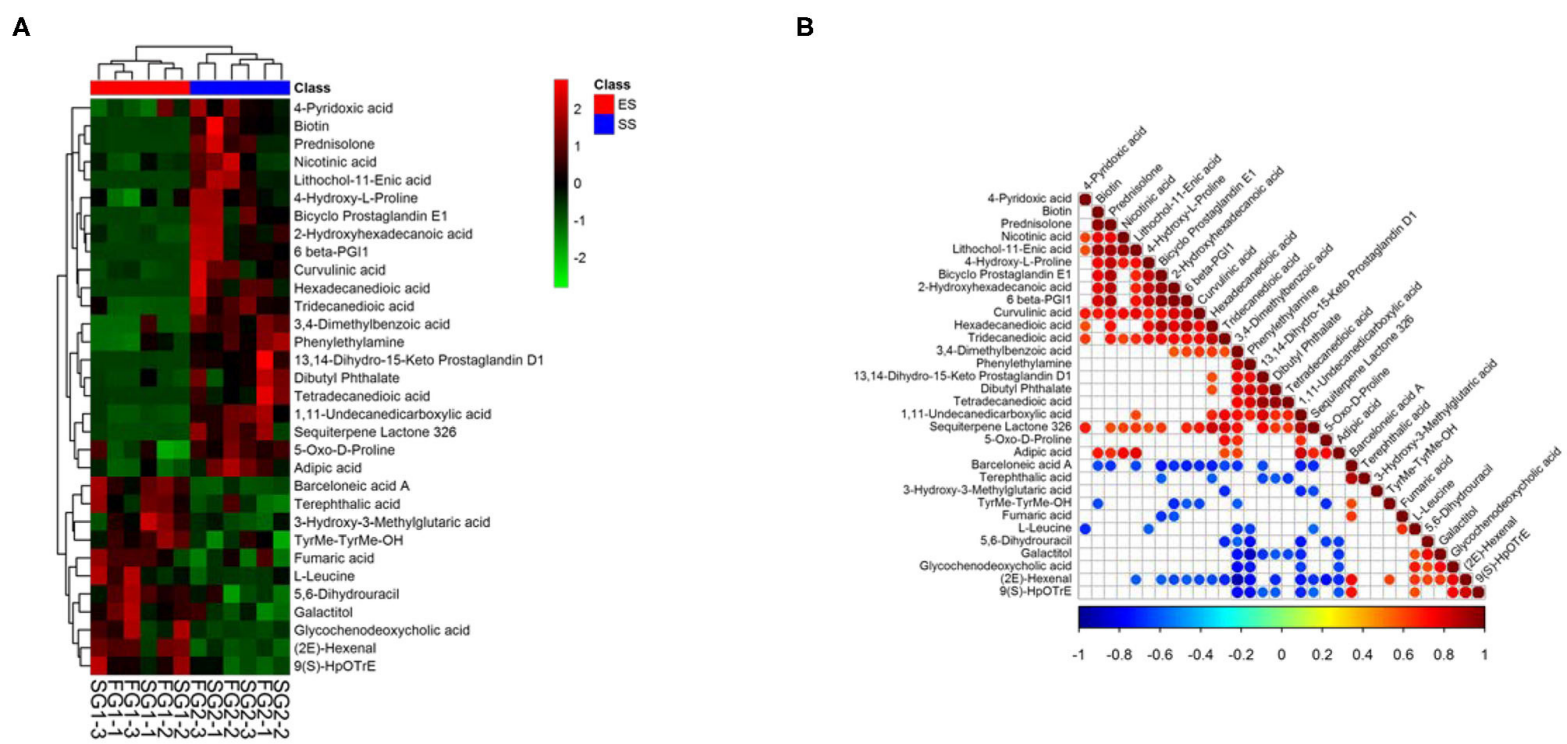
Heatmap showing the significantly different metabolites (VIP > 1.5, **A**) and their co-occurrence patterns **(B)**.

Metabolic biomarker can reveal the significantly different metabolites between young and sub-adult Tibetan sheep. A total of 32 biomarkers were identified between the two groups (Figure 5A). Among these metabolites, 11 metabolite biomarkers were enriched in the lambs, these biomarkers included barceloneic acid A, terephthalic acid, 3-Hydroxy-3-Methylglutaric acid, TyrMe-TyrMe-OH, fumaric acid, L-Leucine, 5,6-Dihydrouracil, galactitol, glycochenodeoxycholic acid, (2E)-hexenal and 9(S)-HpOTrE. In contrast, 21 metabolite biomarkers were more abundant in the sub-adult individuals, these metabolites consisted of 4-Pyridoxic acid, biotin, prednisolone, nicotinic acid, lithochol-11-Enic acid, 4-Hydroxy-L-Proline, bicyclo prostaglandin E1, 2-Hydroxyhexadecanoic acid, 6 beta-PGI1, curvulinic acid, hexadecanedioic acid, tridecanedioic acid, 3,4-Dimethylbenzoic acid, phenylethylamine, 13,14-Dihydro-15-Keto prostaglandin D1, dibutyl phthalate, tetradecanedioic acid, 1,11-Undecanedicarboxylic acid, sequiterpene Lactone 326, 5-Oxo-D-Proline, and adipic acid. In addition, there are complex co-occurrence and co-exclusion patterns in these metabolic biomarkers. For example, 4-Hydroxy-L-Proline was positively correlated with curvulinic acid, while 4-Hydroxy-L-Proline was negatively associated with L-Leucine (Figure 5B).

**Figure 6:**
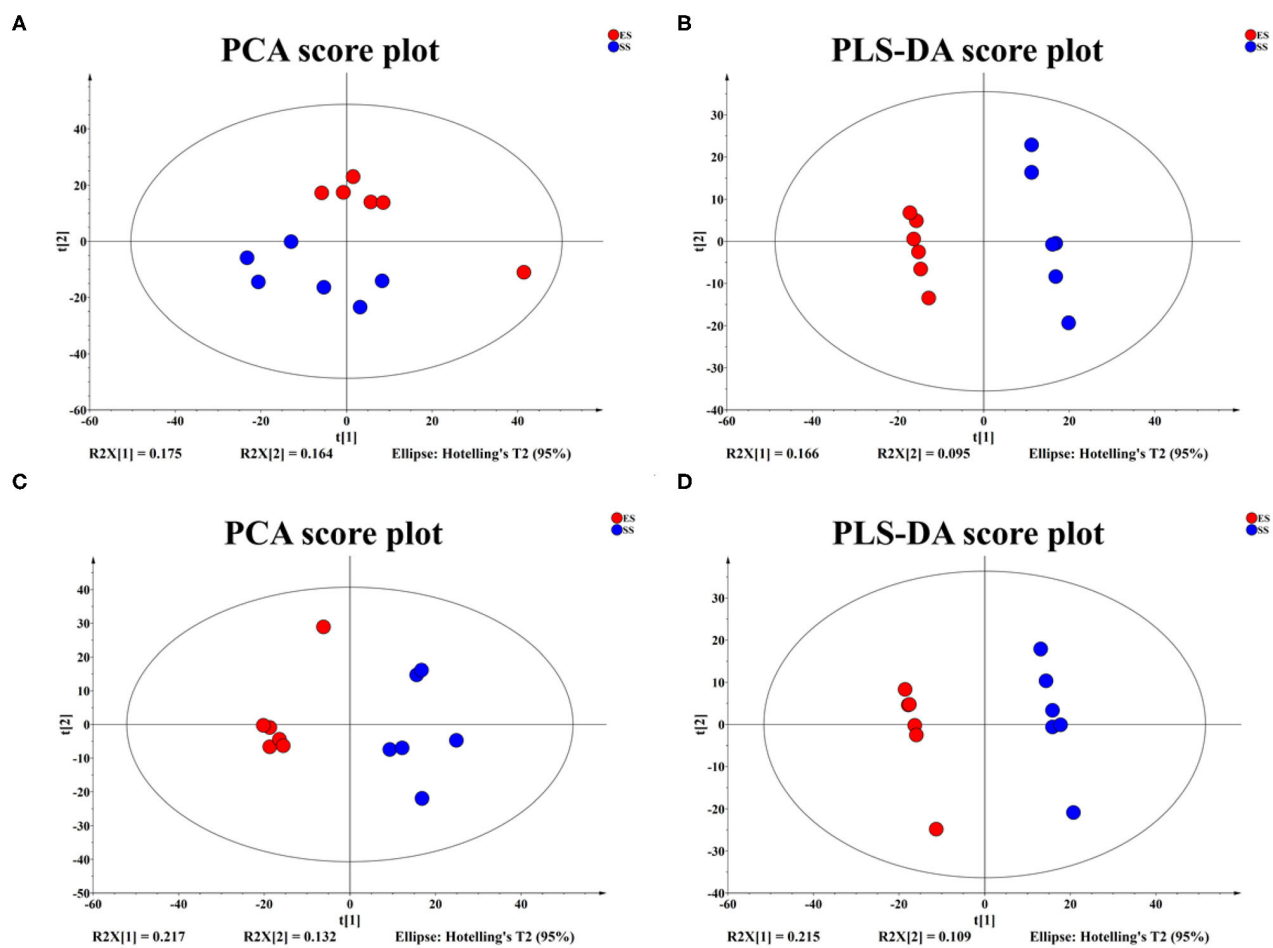
PCA and PLS-DA score plots with positive **(A,B)** and negative **(C,D)** ion modes showing the difference of metabolic profiles between juvenile and sub-adult Tibetan sheep.

**Figure 7:**
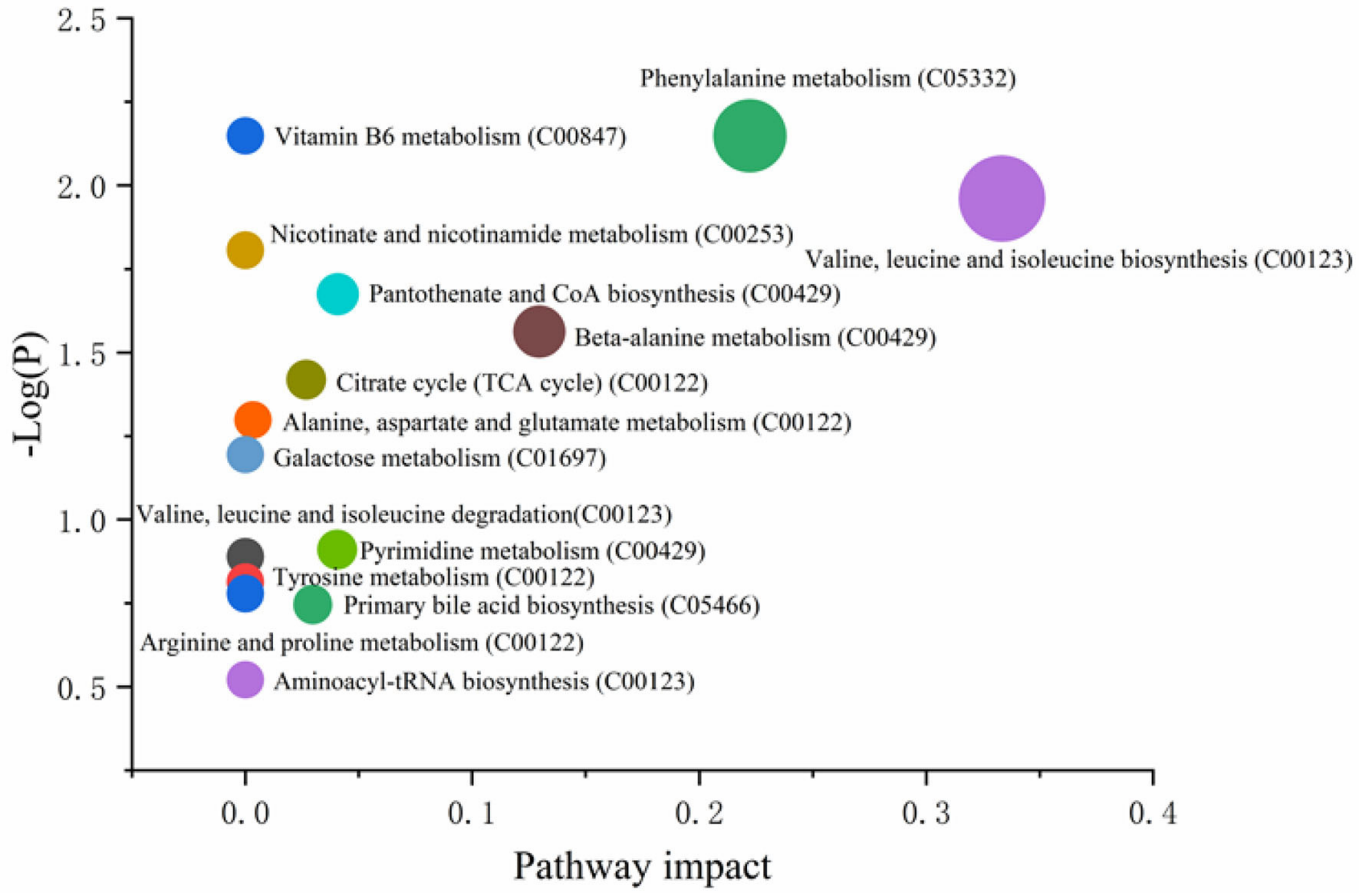
Impacting factor plot of KEEG pathways based on differential metabolites showing the distinct metabolic pathways.

According to the PCA or PLS-DA plots, we found that the young sheep had significantly different metabolomic profiles with sub-adult individuals regardless of ion mode (positive or negative, Figure 6). Based on KEEG pathways analysis, we found that the metabolic biomarkers were involved in 15 functional pathways, such as valine, leucine and isoleucine biosynthesis, phenylalanine metabolism and vitamin B6 metabolis. Among these pathways, the two pathways, including valine, leucine and isoleucine biosynthesis and phenylalanine metabolism, showed the most striking difference between the young and sub-adult sheep (Figure 7). Specifically, L-Leucine that participates in valine, leucine and isoleucine biosynthesis was more abundant in the young sheep. In contrast, phenylethylamine that takes part in phenylalanine metabolism was more enriched in the sub-adult sheep.

### The Difference of Rumen SCFAs Between Young and Sub-adult Sheep

**Figure 8:**
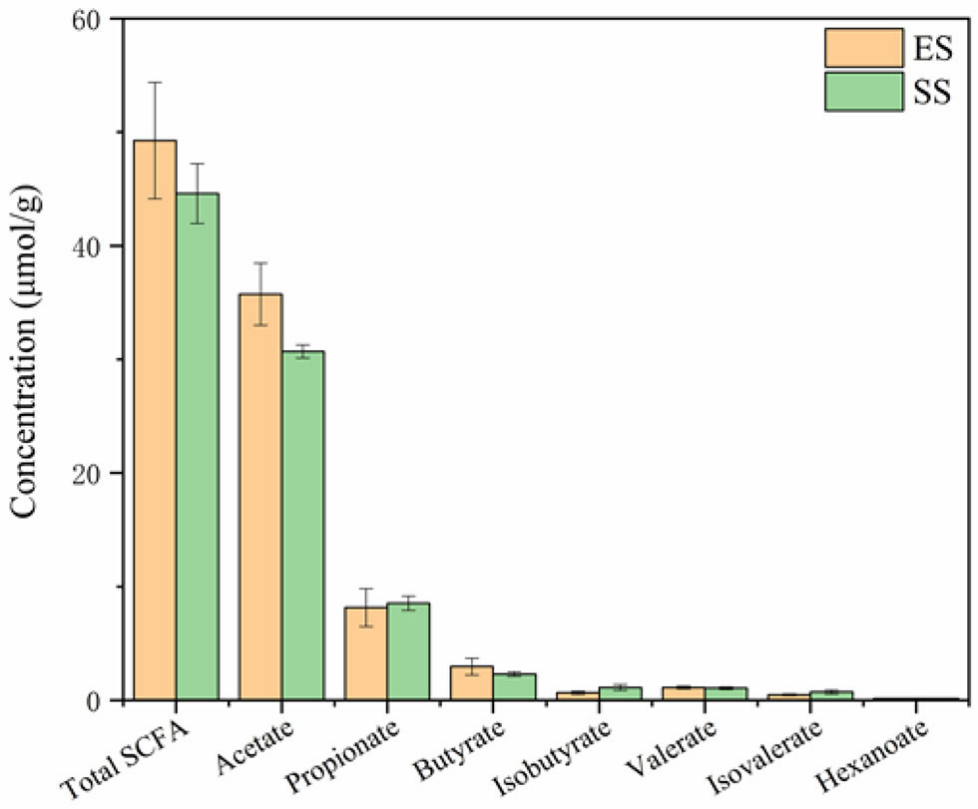
The comparison of short chain fatty acids (SCFAs) in rumen between juvenile and sub-adult Tibetan sheep. One-way ANOVA with Tukey's *post-hoc* test was used to detect the difference. There are no significant difference between the two age stages (*P* > 0.05).

The concentration of acetate, propionate, butyrate, isobutyrate, valerate, isovalerate, hexanoate and total SCFAs were measured in the rumen contents of Tibetan sheep. Acetate, propionate and butyrate were the three most abundant SCFAs in the Tibetan sheep. However, we found that these SCFAs showed no significant difference between the young and sub-adult sheep (*P* > 0.05, Figure 8).

### The Relationship Between Rumen Microbiota, Metabolomic Profiles and SCFAs

**Figure 9:**
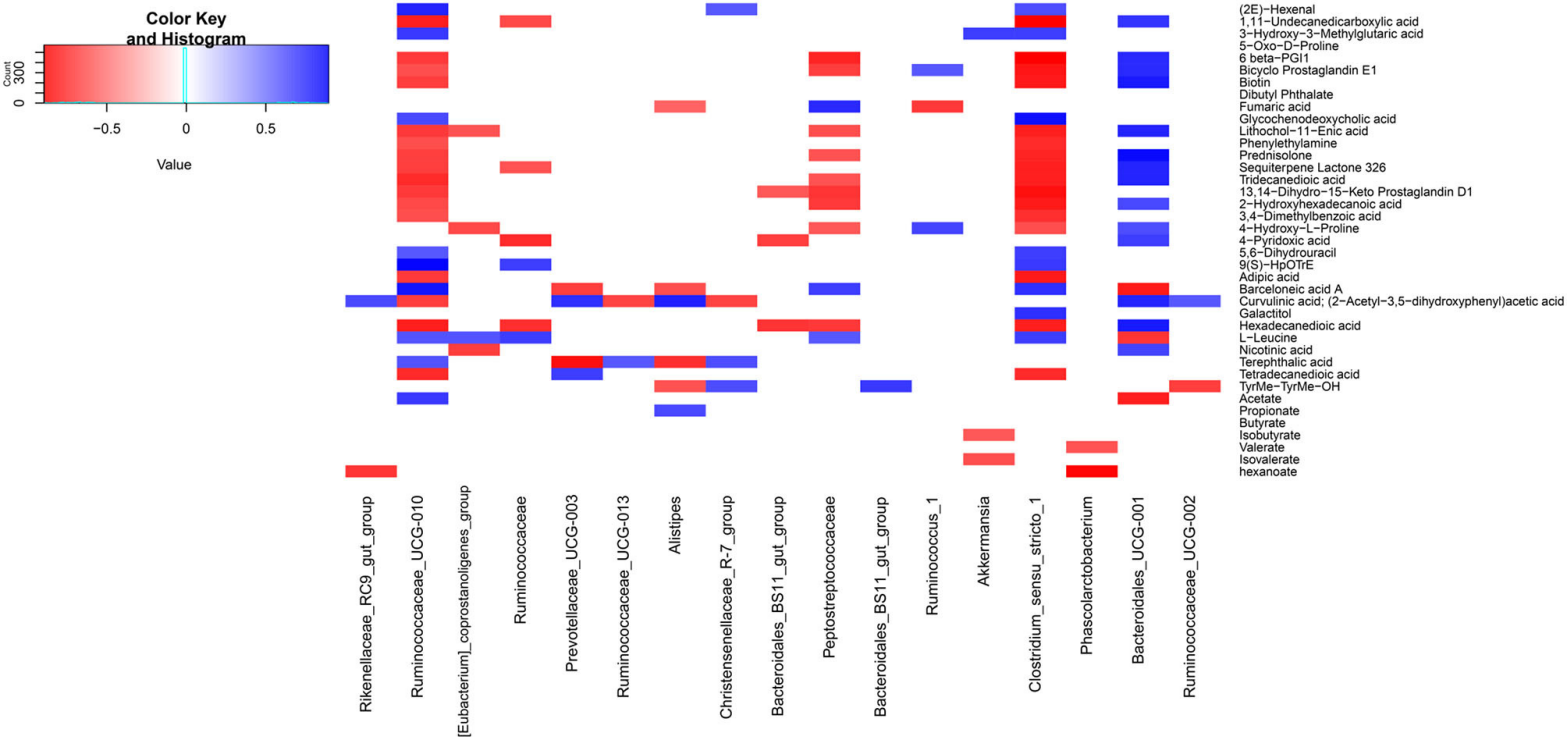
Heatmap plot of spearman correlations between main genera and metabolite biomarkers. Only those correlations with *P* < 0.05 are shown.

Some rumen microbes were associated with metabolites (Figure 9). For example, *Akkermansia* showed positive correlations with 3-Hydroxy-3-Methylglutaric acid, while was negatively associated with isobutyrate and isovalerate. *Ruminococcus*_1 was positively associated with bicyclo prostaglandin E1 and 4-Hydroxy-L-Proline, while showed negative correlations with fumaric acid. *Clostridium*_sensu_stricto_1 was positively correlated with (2E)-Hexenal, 3-Hydroxy-3-Methylglutaric acid, glycochenodeoxycholic acid, 5,6-Dihydrouracil, 9(S)-HpOTrE, barceloneic acid A, galactitol, and L-Leucine, while this genus showed negative correlations with 16 metabolites (e.g., 1,11-Undecanedicarboxylic acid and phenylethylamine).

Mantel test was also used to detect the relationship between rumen microbiota (using weighted UniFrac distance) and metabolomic or SCFA profiles. We found that rumen microbial communities were significantly correlated with metabolomic profiles (positive ion mode, *r* = 0.296, *P* = 0.028; negative ion mode, *r* = 0.343, *P* = 0.019). In addition, SCFA profiles also showed significant associations with rumen microbiota (*r* = 0.315, *P* = 0.029).

## Discussion

Most of previous reports on ruminants only focused on rumen microbiome, but lacked functional and metabolic information. Here, we investigated the relationship among age, rumen microbiome, metabolomics and SCFA profiles. Our data indicate that age significantly influence the rumen microbiome and metabolomic profiles, but had no impacts on SCFA concentrations. In addition, we found that rumen microbial communities were significantly correlated with metabolomic profiles and SCFAs. These results have important significance for understanding nutrition and metabolism of ruminants on the Qinghai Tibetan Plateau.

### The Gut Microbiota in Sub-adult Tibetan Sheep Was More Diverse and Stable Than That in Lambs

The rumen microbial diversity in the sub-adult Tibetan sheep was more diverse than that in the young individuals. This finding is consistent with those of previous studies (5, 26), which showed that the rumen microbial diversity increase with age before adulthood. When ruminants reach close to adulthood, the rumen microbial diversity is relatively stable (5). Compared with the lambs, the higher alpha diversity in the sub-adult sheep may help hosts degrade those indigestible plant polysaccharides and improve rumen performance. It has been demonstrated that gut microbial diversity was positively correlated with cellulolytic activity (25), and also promoted dietary fiber intake (27). Thus, the sub-adult individuals need to better utilize the nutrition from plant-based food by increasing bacterial species.

In addition, the community structures of rumen microbiota were also significantly divergent between the young and sub-adult Tibetan sheep. These results were consistent with those in previous studies (5, 26), which indicated that the microbial community structures were significantly different across ages. The dietary difference between the two age stages may be one important factor impacting the rumen microbial structures. In this study, the sub-adult individuals were mainly fed grass, which consists of plant cellulose. While the young individuals mainly sucked milk, which consists of animal protein. Plant cellulose and animal protein may lead to different microbiota structures (28, 29). In addition, age is also an important impacting factor for the microbial structure. The effects of age on rumen bacterial communities have been demonstrated in Jiao et al. (26) and Jami et al. (5). However, our results can not conclude that whether diet or age was more important in shaping rumen microbiota structures, because these two factors are confounding. Future research should control one factor in order to study the effect of single factor on rumen microbial communities.

Our data showed that Bacteroidetes were more enriched in the sub-adult individuals, while Firmicutes were more abundant in the young sheep. This finding was partly consistent with that in Jami et al. (5), who found that Bacteroidetes and Firmicutes showed an increase trend in relative abundance with age in ruminants. At genus level, *Treponema, Ruminococcus* and several unknown genera from Ruminococcaceae and Spirochaetaceae were enriched in the sub-adult Tibetan sheep. Some members of these bacterial taxa were involved in the degradation of plant cellulose, hemicellulose, and chitin (30). At OTU level, those microbes belonging to *Barnesiella, Akkermansia* and *Eubacterium* were enriched in the young sheep. Some members of *Barnesiella* have also been isolated from human feces (31), and are associated with host immune response. For example, the strain *Barnesiella intestinihominis* may activate anti-cancer immune response of spleen T cell, and thus inhibit tumor growth and improve chemotherapy effect (32). *Eubacterium*, especially *Eubacterium hallii*, may increase fecal butyrate concentrations and modify bile acid metabolism. More importantly, it can improve the insulin sensitivity in diabetic mice, thus contributing potential benefits to animal health (33). *Akkermansia* is mucin-degrading bacteria, and may protect hosts against pathogen invasion. The abundance of this genus in gut microbiome is positively correlated with health and metabolic ability of hosts (34, 35), thus may be developed as potential probiotics in future.

### Rumen Essential Amino Acids and Related Gene Functional Pathways Were Different Between Age Stages, but SCFA Concentration Was Relatively Stable

Our data showed that the metabolomics profiles were significantly different between the young and sub-adult Tibetan sheep based on PCA or PLS-DA analysis, indicating that age is likely to influence the metabolic profiles of animals. These results were consistent with those in Li et al. (25), who also found that age shaped the metabolome of ruminant animals (36). In addition to age, food is also an important impacting factor for the metabolomic difference. Notably, a total of 32 metabolic biomarkers had significantly difference between the two age stages. Among these metabolites, L-Leucine was an essential amino acid and were enriched in the young Tibetan sheep. The animals were unable to synthesize the L-Leucine themselves, and the milk for the young individuals is possibly the main source. In addition to the nutritional value, L-Leucine can also improve the intestinal development (37) and inhibit excessive accumulation of intestinal fat (38), thus contributing to a lot of benefits for the young Tibetan sheep. By KEEG analysis, we found that the functional pathways, valine, leucine and isoleucine biosynthesis, were more abundant in the young individuals, suggesting that the importance and requirement of L- Leucine for the lambs. In contrast, phenylethylamine that takes part in phenylalanine metabolism was more enriched in the sub-adult sheep. Phenylethylamine is also one of the essential amino acids for animals. Most of them are able to oxidized to tyrosine by the phenylethylamine hydroxylase, and together with tyrosine, they may synthesize important neurotransmitters and hormones, which are involved in glucose metabolism and fat metabolism (39). To sum up, metabolomics results demonstrated that the nutritional requirement of essential amino acids were different for the young and sub-adult Tibetan sheep.

Several organic acid were more abundant in the young sheep, such as barceloneic acid A, terephthalic acid, 3-Hydroxy-3-Methylglutaric acid, fumaric acid, and glycochenodeoxycholic acid. These organic acids probably play important roles in host development and metabolism. For example, it has been reported that terephthalic acid was tightly correlated with the ruminal development of lambs (40). Glycochenodeoxycholic acid participates in bile acid metabolism, and its absence in human bile was an indication of cholestasis (41). By contrast, the rumen of sub-adult Tibetan sheep had more content in 11 organic acids, such as 4-Pyridoxic acid, nicotinic acid, lithochol-11-Enic acid, 2-Hydroxyhexadecanoic acid, curvulinic acid, hexadecanedioic acid, tridecanedioic acid, 3,4-Dimethylbenzoic acid, tetradecanedioic acid, 1,11-Undecanedicarboxylic acid, and adipic acid. Among these organic acids, some can improve animal growth and milk yield. For example, nicotinic acid may improve utilization of feed protein and also milk production (42–44). However, the functional roles of these organic acids in rumen need to be further studied. In addition, we found that the co-occurrence patterns of some metabolic products. Whether these metabolites are produced directly by same microorganisms, or indirectly by different microbes, also needs to be explored in future.

SCFAs are the end metabolic products of plant cellulose and other dietary polysaccharides via rumen microbial fermentation. These metabolites contributes to energy and nutrition of ruminants. For example, butyrate can supply ~60–70% energy for normal epithelial cells (45). Besides, these SCFAs may also influence animal physiology, including the synthesis of macromolecular substances *in vivo*. For instance, acetate can been transported to peripheral tissues, and then to liver for participating in cholesterol synthesis (46). Propionate is mainly assimilated by liver, and is involved in gluconeogenesis and protein synthesis (47). In addition, SCFAs also have antimicrobial and anti-inflammatory properties. For example, it has been demonstrated that butyrate, propionate and hexanoate can reduce *Staphylococcus aureus* internalization into bovine mammary epithelial cells and regulate antimicrobial peptide gene expression (48, 49). Our results showed that the concentration of various SCFAs was relatively stable in different age stages, indicating that SCFAs were not impacted by age although gut microbiota changed.

### Rumen Microbiota Was Associated With Metabolomics and SCFAs

Our results showed that rumen microbiota structure was associated with metabolic profiles. This result was congruent with that in goat (7), fish (50), human (51), gorillas (52), and mice (53), suggesting that a tight link between microbiome and metabolome independent of host species. In addition, a weak correlation was also found between rumen microbiota and SCFA profiles, and this finding was consistent with that in our previous study on pika gut microbiome (25). Spearman correlation analysis revealed the potential relationship between rumen microbiota and metabolome or SCFAs. Because some rumen microbes always co-occur with metabolites, this result has important significance for regulating animal nutrition and health by microbiome intervention. However, it seems that we can not conclude which specific microbes responsible for certain metabolites due to the complex interaction among microorganisms, such as resource competition (54) and cross-feeding (55). Thus, future research should isolate these bacterial strains and study the relationship between specific bacteria and metabolites.

## Conclusion

In conclusion, we found that both rumen microbial community structures and metabolite profiles of Tibetan sheep were distinct at different ages, but rumen SCFA concentration was relatively stable between the two age stages. Notably, metabolomics analysis showed that the nutritional requirement of essential amino acids in rumen were different between the lambs and sub-adult Tibetan sheep. We found L-Leucine that participates in valine, leucine and isoleucine biosynthesis was more abundant in the lambs, while phenylethylamine that takes part in phenylalanine metabolism was enriched in the sub-adults. In addition, rumen microbita was associated with metabolomics and SCFAs, indicating the close relationship between microbial composition and metabolites. These results have important significance for regulating animal nutrition and health by microbiome intervention.

## Data Availability Statement

All the original 16S rRNA gene data in this study were submitted to the European Nucleotide Archive, and were available by accession no. PRJEB36249.

## Ethics Statement

Animal ethics approval for the present project was obtained from the Animal Ethics Committee of Lanzhou University.

## Author Contributions

HL and JQ designed this study. JQ carried out the animal experiments and sample collection. HL was responsible for the molecular experiments, data analysis, and wrote the original manuscript. All authors revised and edited the manuscript.

## Conflict of Interest

The authors declare that the research was conducted in the absence of any commercial or financial relationships that could be construed as a potential conflict of interest.

## Abbreviations

SCFA: Short chain fatty acid
OTU: Operational taxonomic units
PCoA: Principal coordinates analysis
RDP: Ribosomal Database Project.
